# pH-Dependent Protein Binding Properties of Uremic Toxins In Vitro

**DOI:** 10.3390/toxins13020116

**Published:** 2021-02-04

**Authors:** Suguru Yamamoto, Kenichi Sasahara, Mio Domon, Keiichi Yamaguchi, Toru Ito, Shin Goto, Yuji Goto, Ichiei Narita

**Affiliations:** 1 Division of Clinical Nephrology and Rheumatology, Niigata University Graduate School of Medical and Dental Sciences, Niigata 951-8510, Japan; mio.dmn@gmail.com (M.D.); itotoru.gt@gmail.com (T.I.); gotos@med.niigata-u.ac.jp (S.G.); naritai@med.niigata-u.ac.jp (I.N.); 2 Institute for Protein Research, Osaka University, Yamadaoka 3-2, Suita, Osaka 565-0871, Japan; ksasahara@protein.osaka-u.ac.jp (K.S.); kyamaguchi@protein.osaka-u.ac.jp (K.Y.); gtyj8126@protein.osaka-u.ac.jp (Y.G.)

**Keywords:** pH, uremic toxins, albumin, indoxyl sulfate, isothermal titration calorimetry

## Abstract

Protein-bound uremic toxins (PBUTs) are difficult to remove using conventional dialysis treatment owing to their high protein-binding affinity. As pH changes the conformation of proteins, it may be associated with the binding of uremic toxins. Albumin conformation at pH 2 to 13 was analyzed using circular dichroism. The protein binding behavior between indoxyl sulfate (IS) and albumin was examined using isothermal titration calorimetry. Albumin with IS, and serum with IS, p-cresyl sulfate, indole acetic acid or phenyl sulfate, as well as serum from hemodialysis patients, were adjusted pH of 3 to 11, and the concentration of the free PBUTs was measured using mass spectrometry. Albumin was unfolded at pH < 4 or >12, and weakened interaction with IS occurred at pH < 5 or >10. The concentration of free IS in the albumin solution was increased at pH 4.0 and pH 11.0. Addition of human serum to each toxin resulted in increased free forms at acidic and alkaline pH. The pH values of serums from patients undergoing hemodialysis adjusted to 3.4 and 11.3 resulted in increased concentrations of the free forms of PBUTs. In conclusion, acidic and alkaline pH conditions changed the albumin conformation and weakened the protein binding property of PBUTs in vitro.

## 1. Introduction

Patients with chronic kidney disease (CKD) undergoing dialysis treatment have worse clinical outcomes owing to various systemic disorders including cardiovascular disease, mineral and bone disorders, and infectious disease. One of the reasons underlying the development of these outcomes is the direct or indirect interactions between various uremic toxins and organ/tissues [1,2,3,4]. Among them, several solutes, such as indoxyl sulfate (IS) and p-cresyl sulfate (PCS), bind easily to large proteins including albumin and are called protein-bound uremic toxins (PBUTs) [5]. Basic and clinical studies have demonstrated the strong toxicities of PBUTs which are associated with systemic disorders [1,2,3,4]; however, these solutes are difficult to remove with conventional hemodialysis treatment owing to their high protein binding property [6]. Thus, the development of a method that increases the removal of PBUTs should be the most fundamental approach to improving clinical outcomes in patients undergoing dialysis. One of the possible therapeutic strategies will be to weaken the binding property of PBUTs because the increase in the free form of uremic toxins will facilitate the removal through dialysis treatment.

Previous basic studies have identified the uremic toxin-binding site of albumin in vitro [7,8,9,10]. Albumin molecules change their conformations with the change of pH in the solution [8,11,12]. Thus, the pH-induced conformational change of proteins may be associated with the binding property of PBUTs. In this study, we examined the protein-binding properties of uremic toxins with pH changes in vitro.

## 2. Results

### 2.1. Conformational Change of Albumin Depending on the pH of Solutions

**Figure 1:**
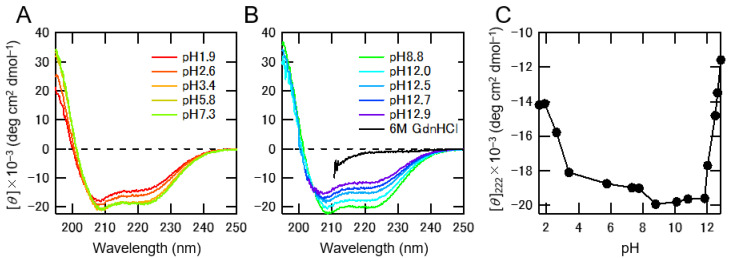
pH-dependent conformational change of albumin. Representative Circular dichroism (CD) spectra of albumin in acidic pH (**A**) and basic pH (**B**) regions. The pH of solutions is indicated within the figures. The spectrum of completely unfolded albumin in 6 M GdnHCl is indicated as a black line (**B**). (**C**) The ellipticities of CD spectra measured at 222 nm are plotted against the pH of the solutions.

To gain insight into the pH-dependent conformational change of albumin, we measured Circular dichroism (CD) spectra at various pH conditions. At around neutral pH, CD spectra exhibited double minima at 208 nm and 222 nm (Figure 1A,B), indicating that albumin had an α-helical conformation. We then plotted the ellipticities at 222 nm (Figure 1C), which were correlated with the α-helical content of albumin and the pH of the solutions. The ellipticities of CD spectra decreased under both acidic and alkaline pH conditions, indicating that albumin unfolded at pH below 5 or above 12, as observed previously [12].

### 2.2. Interaction between IS and Albumin Depending on the pH of the Solution

**Figure 2:**
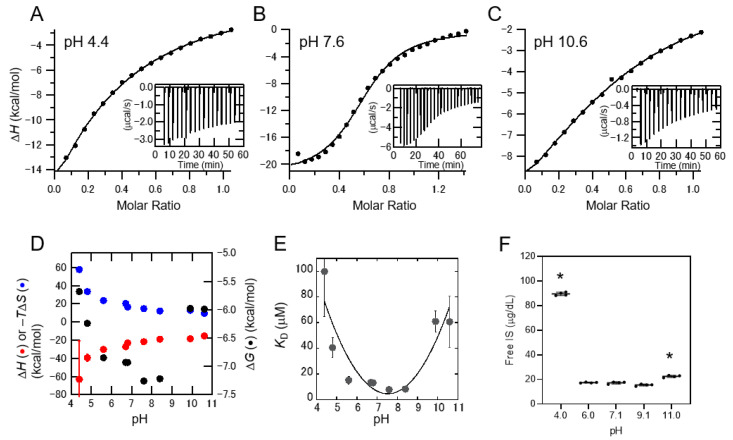
Interaction between indoxyl sulfate (IS) and albumin as monitored using Isothermal Titration Calorimetry. (**A**–**C**) Total enthalpy change (Δ*H*) for each injection at pH 4.4 (**A**), 7.6 (**B**), and 10.6 (**C**). Insets show the direct enthalpy change resulting from the addition of IS to 150 μM albumin. (**D**, **E**) Thermodynamic parameters (Δ*H* (red), –*T*Δ*S* (blue), and Δ*G* (black)) (**D**) and dissociation constant (*K*_D_) (**E**) are plotted against pH of solutions. (**F**) Protein-unbound IS at pH 4–11 in the presence of albumin. pH of albumin solution (3 g/dL) adjusted to 4.0, 6.0, 7.1, 9.1, and 11.0, and IS (2 mg/dL) was added to each albumin solution and the concentrations of free-form IS were measured. Data are shown as the mean ± standard deviation of four independent experiments. * *p* < 0.01 vs. pH 7.1.

To examine the affinity between IS and albumin, we performed Isothermal Titration Calorimetry (ITC) experiments under various pH conditions. Negative peaks resulting from the reaction indicated the exothermic nature of the binding process (Figure 2A–C). At pH 7.6, the titration of IS with albumin showed a saturation curve (Figure 2B), with the strength of the interaction at pH 7.6 being stronger than that at pH 4.4 (Figure 2A) and pH 10.6 (Figure 2C). Complete saturations were achieved at a molar ratio of >1 under all conditions. The enthalpy change (Δ*H*), entropy change (–*T*Δ*S*), and free energy change (Δ*G*) for binding were plotted against the pH of the solutions (Figure 2D). The value of Δ*H* negatively correlated with the pH of the solution, where negatively charged IS could interact with positively charged albumin (isoelectric point of albumin was 4.7). However, the Δ*G* that resulted from the sum of Δ*H* and –*T*Δ*S* decreased at neutral pH. We then plotted the dissociation constants (*K*_D_) against the pH of the solutions (Figure 2E). Although the *K*_D_ was less than 10 μM at neutral pH, it significantly increased at a pH lower than 5 or higher than 10. These pH regions (pH < 5 or >10) were almost consistent with the partially unfolded albumin in the CD experiments, indicating that the interaction between IS and albumin was weakened by the partially unfolded albumin.

The pH of human serum albumin solution (3 mg/dL) was adjusted to 4.0–11.0, and IS at a concentration of 2 mg/dL was added to reveal the protein-binding property of IS for various pH conditions. It was observed that the same concentration of total IS was present in all the solutions. (Figure S1A). The concentration of free IS significantly increased at pH 4.0 and pH 11.0 (pH 4.0: 89.49 ± 1.38 μg/dL and pH 11.0: 22.45 ± 1.38 μg/dL vs. pH 7.1: 17.20 ± 0.87 μg/dL, *p* < 0.01, respectively, Figure 2F and Table S1). These results suggest that acidic and alkaline pH conditions weakened the interaction between IS and albumin.

### 2.3. pH-Dependent Protein-Binding Property of Uremic Toxins in Serum

**Figure 3:**
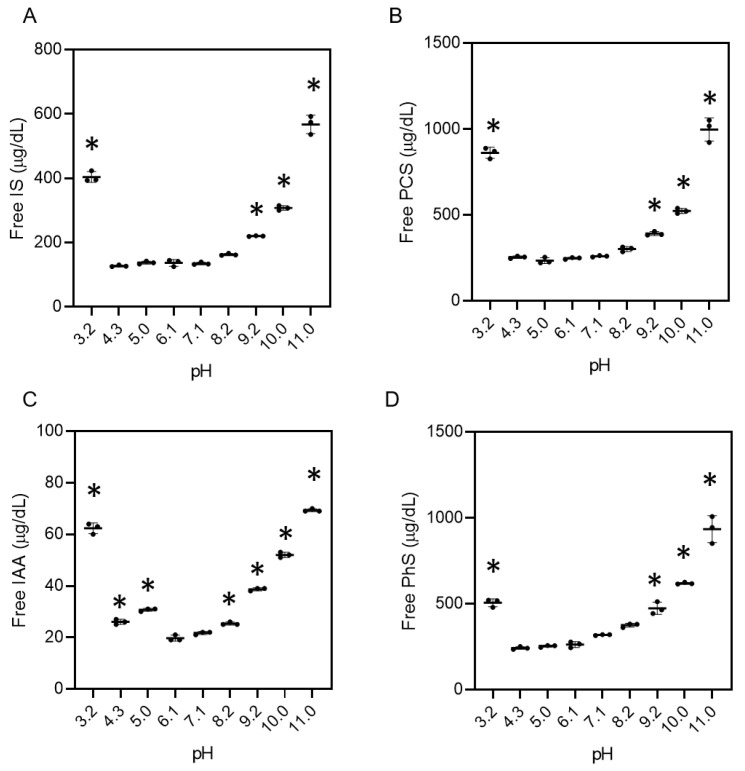
Protein-unbound uremic toxins at pH 3.2–11.0 in human serum. Human serum from non-chronic kidney disease patients containing indoxyl sulfate (IS) (**A**), p-cresyl sulfate (PCS) (**B**), indole acetic acid (IAA) (**C**), or phenyl sulfate (PhS) (**D**) at the typical concentrations of patients undergoing dialysis was adjusted pH from 3.2 to 11.0. The concentrations of free-form uremic toxins were measured. Data are shown as the mean ± standard deviation of three independent experiments. * *p* < 0.01 vs. pH 7.1.

When human serum from non-CKD patients adjusted from pH 3.2 to 11.0 was added to the IS at a concentration typically found in dialysis patients (Figure S2A), the concentrations of the free form of the IS in the serum under acidic and alkaline pH conditions was higher than that in serum at a neutral pH (pH 3.2: 403.3 ± 16.7 μg/dL, pH 9.2: 220.0 ± 1.0 μg/dL, pH 10.0: 307.0 ± 7.9 μg/dL, pH 11.0: 567.4 ± 28.6 μg/dL vs. pH 7.1: 134.7 ± 3.5 μg/dL, *p* < 0.01, Figure 3A and Table S2). When PCS, indole acetic acid (IAA), and phenyl sulfate (PhS) were reacted with serum from non-CKD patients under various pH conditions, the same trends were observed (Figure 3B–D and Figure S2B–D, and Table S2).

**Figure 4:**
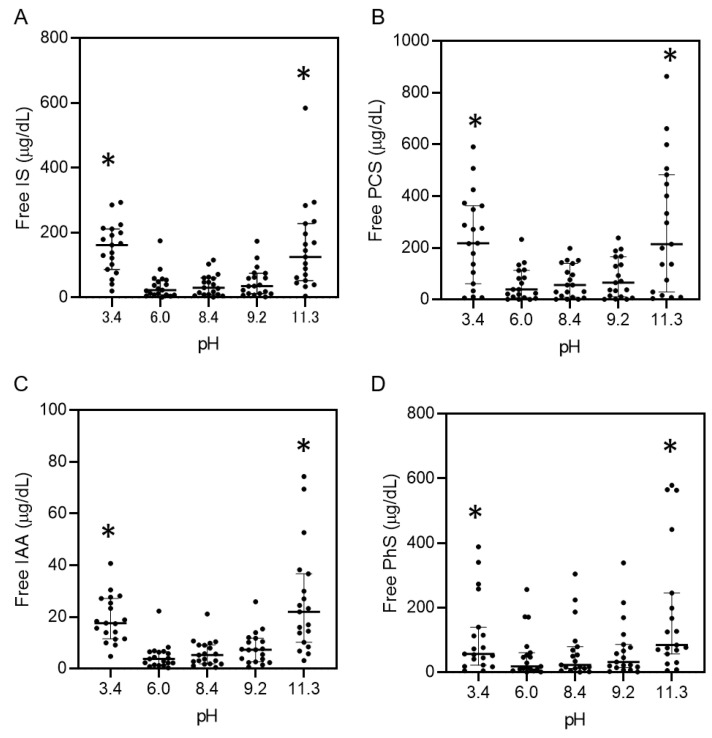
Protein-unbound uremic toxins at pH 3.4–11.3 in serum specimens from patients undergoing hemodialysis treatment. Serum levels of free indoxyl sulfate (IS) (**A**), p-cresyl sulfate (PCS) (**B**), indole acetic acid (IAA) (**C**), or phenyl sulfate (PhS) (**D**) at each pH were measured. Data are expressed as the median values (interquartile range) of 19 individuals. * *p* < 0.01 vs. pH 8.4.

The protein-binding property of uremic toxins in serum from patients undergoing dialysis was also assessed under various pH conditions. Table S3 shows the data of 19 patients undergoing maintenance hemodialysis which are typical characteristics of patients with CKD undergoing dialysis treatment. The pH of serum not adjusted with the buffer was 7.9 ± 0.1. The pH of serum from the patients was adjusted from 3.4 to 11.3 with the buffer, with the same volume with serum, which showed the same concentrations of total uremic toxins in all the solutions (Figure S3A,C,E,G). The concentrations of the free forms of IS, PCS, PhS, and IAA increased under acidic and alkaline pH conditions (e.g., IS: pH 3.4, 152.5 ± 77.6 μg/dL; pH 11.3, 153.8 ± 135.5 μg/dL vs. pH 8.4, 38.8 ± 33.4 μg/dL; *p* < 0.01, Figure 4 and Table S4) as well as the protein-bound rate (e.g., IS: pH 3.4, 83.3 ± 5.4%; pH 11.3, 84.3 ± 11.7% vs. pH 8.4, 96.5 ± 2.4%; *p* < 0.01, Figure S3B,D,F,H). We also examined pH-induced protein-bound properties of hippuric acid (HA) and creatinine, which are known to have lower protein-binding properties than IS, PCS, PhS, and IAA. As expected, the acidic and alkaline pH did not change the concentration (Figure S4).

These results suggest that acidic and alkaline pH conditions increased the free form of uremic toxins in serum and simultaneously weakened the protein-binding properties of PBUTs.

## 3. Discussion

In this study, we showed that acidic and alkaline pH conditions weakened the protein-binding affinity of PBUTs in vitro. One of the key factors for the reduced binding affinities of PBUTs is the pH-induced conformational change of proteins.

Removal of PBUTs using conventional dialysis treatment is insufficient. For example, circulating IS and PCS show a high protein-bound rate (97.7% and 95.1%), with reduction rates of merely 31.8% and 29.1%, respectively, using standard hemodialysis [6]. Several studies have suggested that direct hemoperfusion using activated carbon [13], oral charcoal administration [14] as well as adding activated charcoal into the dialysate [15] are able to reduce the serum level of PBUTs, and the adsorption method may be one of the attractive strategies to remove PBUTs. Another possible mechanism is through increasing the free PBUTs with weakened protein-binding property. Previous studies have detected binding sites (sites 1 and 2) in albumin for several uremic toxins [7,8,9,10,16,17], and IS and PCS are known to be competitive binding inhibitors because the binding site of albumin is the same for both substances [18]. In this study, our focus was on the effect of pH conditions on the conformational changes in proteins. In fact, acidic and alkaline pH conditions induced the conformational change of albumin toward unfolded (Figure 1), and such pH conditions weakened the binding property of albumin with IS (Figure 2). Since the pH-induced conformational change of albumin corresponds to the increase in free-form uremic toxins, the affinity of albumin binding sites to uremic toxins also weakened. The same results were observed in the serum from non-CKD patients with uremic toxins as well as the serum from patients undergoing hemodialysis treatment (Figure 3 and Figure 4) which contained various kinds of proteins. These data suggest that several kinds of uremic toxins bind to proteins ubiquitously, and their affinities are affected by pH conditions in the serum owing to the conformational change of the proteins. In the calorimetric analysis shown in Figure 2, albumin with conformational abnormalities at acidic and alkaline pH values interacted less with IS in vitro than the other substance. In contrast, when the pH of the uremic serum changed under acidic and alkaline conditions, uremic toxins were separated from the proteins (Figure 4). These results suggest that conformational changes in albumin at acidic and alkaline pH are associated with both binding and dissociation with uremic toxins. In our study, the data showed different trends for the protein-biding properties for uremic toxins between the albumin solution (Figure 2F), non-CKD serum (Figure 3), and uremic serum (Figure 4). For example, the free IS level in non-CKD serum at pH 4.3 was not higher than that at pH 7.1 (Figure 3A) while albumin at pH 4.0 showed less protein-binding property with IS than that at pH 7.1 (Figure 2F). This discrepancy may be induced with the difference of setting between the albumin solution and serum containing various kinds of proteins. Another possibility may be the effect of the isoelectoric point of albumin (pH 4.7) on the protein-binding property with uremic toxins. It may enhance the interaction with uremic toxins even when the protein conformation was changed at acidic pH. In this study, PhS showed the weakest reaction for the pH-induced binding ability compared to other PBUTs (Figure 4). Previous studies have suggested that IS, IAA, and PCS bound to site 2 in the albumin via hydrogen bonds [9,17], while little information is reported for PhS. Thus, the binding site of PhS, or the intermolecular bonding interaction may be different with other uremic toxins. Further studies are needed to elucidate the detailed characteristics of each uremic toxin for its respective protein-binding properties. Our calorimetric analysis (Figure 2) suggested that the binding of albumin to IS was dose-dependent and that the binding of IS to albumin was the same at both low and high doses. These data suggest that low concentrations of IS in early-stage CKD patients show the same trend of protein-binding affinity as that in end-stage kidney disease patients. Albumin in the blood combines with not only uremic toxins but also with various molecules, such as drugs and fatty acids, and the changing conformation of protein should be considered in pharmacological effects in clinical use.

This is an in vitro study, but the insights gained may lead to the development of urgently needed therapeutic strategies to increase the removal of PBUTs in dialysis treatment. When the pH conditions of blood are modified through some external stimulations while passing through the dialyzer column, increased removal of free uremic toxins could be achieved using a diffusion/convection method. In a basic study, pigs with hepatic injury and sepsis were treated with a blood purification system using circulating albumin dialysate [19]. Before the albumin dialysate reached the filters, acid (HCl) or base (NaOH) was added for toxin removal from albumin. These systems may be more effective in increasing the removal of PBUTs in hemodialysis patients if huge changes in the pH of serum during passing through the dialyzer column can be realized. There are limitations to apply our data to the current clinical dialysis treatment because huge pH changes in human serum are reported only in vitro study [20], not in a clinical study. However, we would like to emphasize the importance of conformational changes of proteins to weaken their binding properties for uremic toxins using not only changing pH but other methods. We believe protein conformational change will be one of important concepts to increase the removal of uremic toxins with blood purification treatment which will lead to better clinical outcomes in patients undergoing dialysis treatment.

## 4. Conclusions

Both acidic and alkaline pH conditions could change the conformation of albumin and increase the amount of free PBUTs in vitro. A weakened protein bound property of PBUTs may increase the removal with hemodialysis treatment, and the modification of protein conformation is one of the effective strategies. The maintenance of a low level of PBUTs with some interventions for protein conformational change will improve clinical outcomes in end-stage kidney disease patients.

## 5. Materials and Methods

### 5.1. CD Measurement of Albumin

CD spectra were recorded with a Jasco spectropolarimeter J-820 (Jasco Co., Ltd., Tokyo, Japan) using a quartz cell with a light path of 1 mm and a protein concentration of 2 μM at 25 °C. The pH of the solution was adjusted using HCl or NaOH. The results were expressed as the mean residue ellipticity [*θ*] (deg cm^2^/dmol).

### 5.2. Interaction between Albumin and Indoxyl Sulfate Monitored by ITC

ITC measurements were performed using a PEAQ-ITC instrument (MicroCal™, Malvern Instruments, Malvern, UK) at various pH conditions and 37 °C. The pH of the solution was adjusted using HCl or NaOH. IS (1.1 mM) in the syringe was titrated into 150 μM of human serum albumin (Sigma–Aldrich Co. St. Louis*,* MO, USA) in the ITC cell. ITC data were analyzed using Origin software (OriginLab Co., Ltd., Northampton, MO, USA).

### 5.3. Reaction of Albumin with Indoxyl Sulfate under Changing pH

The pH of human serum albumin (3 g/dL) in PBS was adjusted to 4.0, 6.0, 7.1, 9.1, and 11.0 using HCl or NaOH. IS (2 mg/dL) (Sigma–Aldrich) in PBS was added to the albumin solutions at each pH, and the mixtures were incubated at 37 °C for 30 min. Concentrations of free and total form IS were measured.

### 5.4. Reaction of Uremic Toxins and Serum from Normal Subjects with Changing pH

IS was added to human serum from non-CKD patients (Cosmo Bio Co., Ltd., Tokyo, Japan) at a concentration of 3.74 ± 0.08 mg/dL, which is typical of dialysis patients [6]. The pH of the serum containing IS was adjusted to 3.2, 4.3, 5.0, 6.1, 7.1, 8.2, 9.2, 10.0, and 11.0 by adding phosphoric acid or NaOH to the same volume of buffer. Concentrations of free and total IS were measured. The same procedures were repeated for PCS (3.64 ± 0.05 mg/dL) (Eiweiss Chemical Co. Ltd., Shizuoka, Japan), IAA (0.153 ± 0.004 mg/dL) (Tokyo Chemical Industry Co., Ltd., Tokyo, Japan), and PhS (1.35 ± 0.02 mg/dL) (Kureha Co., Ltd., Tokyo, Japan).

### 5.5. Uremic Serum from Patients with Changing pH

We recruited 19 patients undergoing maintenance hemodialysis treatment who had no residual kidney function from a single dialysis unit. All eligible patients received 4–5 h of hemodialysis thrice weekly using standard bicarbonate dialysate (Na^+^: 140 mEq/L, K^+^: 2.0 mEq/L, Ca^2+^: 2.75 mEq/L, Mg^2+^: 1.0 mEq/L, Cl^−^: 112.25 mEq/L, HCO_3_^−^ 27.5 mEq/L) and 1.6–2.1 m^2^ dialyzers with synthetic membranes, polysulfone or polyethersulfone. The flow rates for blood and the dialysate were set at 180–250 mL/min and 500 mL/min, respectively. Dialysis adequacy was estimated by using Kt/V_urea_. Serum specimens at pre-dialysis treatment were collected and immediately frozen at −30 °C and thawed just before adjusting the pH. The pH of uremic serum from each patient was adjusted to 3.4 ± 0.2, 6.0 ± 0.2, 8.4 ± 0.2, 9.2 ± 0.2, and 11.3 ± 0.2 using the same volume of buffer (×2 dilution), and the mixtures were incubated at 37 °C for 30 min. Concentrations of uremic toxins in free and total forms were measured. This study adhered to the guidelines of the Declaration of Helsinki and was approved by the Central Ethics Committee at Niigata University (No. 2019-0060, date: 5 June 2019). It has been registered at the University Hospital Medical Information Network Center (UMIN000040111). All participants provided written informed consent.

### 5.6. Measurement of Uremic Toxins

Serum specimens collected from patients and the reaction solutions were immediately frozen at −30 °C and thawed just before the measurement of PBUTs. Respective levels of total and free forms of four PBUTs such as IS, PCS, IAA, and PhS were measured using mass spectrometry as described previously [6].

### 5.7. Statistical Analysis

The data for the reaction between albumin/human serum and uremic toxins were expressed as the mean ± standard deviation. One-way analysis of variance followed by Bonferroni’s multiple comparisons test was used to compare the differences between the groups. Data for the uremic toxins at pH 3.4–11.3 were expressed as medians (interquartile range). Friedman’s test was used to compare the differences between data at pH 8.4 and those at pH 3.4, 6.0, 9.2, and 11.3. The number of biological samples for each group is stated in the corresponding figure legends. Differences were considered statistically significant if the *p*-value was <0.05.

## Data Availability

Data are available upon request, please contact the contributing authors.

## Supplementary Materials

The following are available online at https://www.mdpi.com/2072-6651/13/2/116/s1, Table S1: Concentration of free IS in albumin solution at pH 4.0–11.0. Table S2: Concentrations of free IS, PCS, IAA, and PhS in non-CKD serum at pH 3.2–11.0. Table S3: Demographic and clinical characteristics, Table S4: Concentrations of free IS, PCS, IAA, and PhS in uremic serum at pH 3.4–11.3. Figure S1: Protein-unbound and -bound indoxyl sulfate at pH 4.0–11.0 in the presence of albumin, Figure S2: Protein-unbound and -bound uremic toxins at pH 3.2–11.0 in human non-CKD serum, Figure S3. Protein-unbound and -bound uremic toxins and protein-bound rates at pH 3.4–11.3 in serum from patients undergoing hemodialysis. Figure S4. Serum level of HA and creatinine before and after ultracentrifugation at pH 3.4–11.3.
