# Supplementary material for: pH-Dependent Protein Binding Properties of Uremic Toxins In Vitro
Source: Toxins (Basel). 2021 Feb 4;13(2):116. doi: 10.3390/toxins13020116 (PMC7914709; doi:10.3390/toxins13020116)
Supplement: Supplementary file 1 [file toxins-13-00116-s001.pdf]

Supplementary Materials: pH-Dependent Protein Binding Properties of Uremic Toxins in Vitro

Suguru Yamamoto, Kenichi Sasahara, Mio Domon, Keiichi Yamaguchi, Toru Ito, Shin Goto, Yuji Goto and Ichiei Narita

Table S1. Concentration of free IS in albumin solution at pH 4.0–11.0.

**pH**	**4.0**	**6.0**	**7.1**	**9.1**	**11.0**
Free IS (mg/dL)	89.49 ± 1.38 *	17.43 ± 0.39	17.20 ± 0.87	15.45 ± 0.77	22.45 ± 0.89 *

IS, indoxyl sulfate. * $p < 0.01$ vs pH 7.

Table S2. Concentrations of free IS, PCS, IAA, and PhS in non-CKD serum at pH 3.2–11.0.

pH	3.2	4.3	5.0	6.1	7.1	8.2	9.2	10.0	11.0
Free IS (μg/dL)	403.67 ± 16.77 *	127.00 ± 2.65	137.33 ± 4.51	136.67 ± 10.21	134.67 ± 3.79	162.33 ± 3.21	220.00 ± 1.00 *	307.33 ± 7.51 *	567.33 ± 28.59 *
Free PCS (μg/dL)	861.67 ± 32.04 *	253.67 ± 7.09	233.33 ± 17.21	247.00 ± 5.29	259.33 ± 4.04	301.04 ± 14.42	391.80 ± 11.36 *	522.67 ± 14.57 *	997.00 ± 67.02 *
Free PhS (μg/dL)	505.33 ± 22.86 *	242.00 ± 7.55	253.00 ± 6.08	262.00 ± 16.7	318.33 ± 2.89	375.21 ± 11.27	473.24 ± 35.37 *	619.00 ± 5.29 *	934.00 ± 78.48 *
Free IAA (μg/dL)	62.33 ± 2.08 *	26.00 ± 1.00 *	30.67 ± 0.58 *	19.67 ± 1.15	21.67 ± 0.58	25.33 ± 0.58 *	38.67 ± 0.58 *	52.00 ± 1.00 *	69.33 ± 0.58 *

IAA, indole acetic acid; IS, indoxyl sulfate; PCS, p-cresyl sulfate; PhS, phenyl sulfate. * $p < 0.01$ vs pH 7.1.

Table S3. Demographic and clinical characteristics.

**Demographic and Clinical Characteristics**	
Number of patients	19
Age, years	68.2 ± 10.3
Sex, male/female	10/9
Body mass index, kg/m^2	22.9 ± 4.1
Cause of ESKD, CGN/DM/others	10/5/4
Systolic blood pressure, mmHg	153.9 ± 22.9
**Dialysis**	
Duration of dialysis, months	106.5 ± 132.4
Kt/V_{urea}	1.42 ± 0.34
Laboratory data	
Urea nitrogen, mg/dL	58.6 ± 18.3
Creatinine, mg/dL	9.91 ± 3.33
Albumin, g/dL	3.11 ± 0.55
Blood hemoglobin, g/dL	10.7 ± 1.6
Calcium, mg/dL	9.6 ± 0.8
Phosphorus, mg/dL	5.6 ± 1.5
Intact PTH, pg/mL	166.1 ± 136.4
C-reactive protein, mg/dL	0.71 ± 0.95

CGN, chronic glomerulonephritis; DM, diabetes mellitus; ESKD, end-stage kidney disease; PTH, parathyroid hormone.

Table S4. Concentrations of free IS, PCS, IAA, and PhS in uremic serum at pH 3.4–11.3.

**pH**	**3.4**	**6.0**	**8.4**	**9.2**	**11.3**
Free IS (μg/dL)	161.7 (94.3–205.4) *	22.7 (8.2–51.2)	30.1 (9.5–59.4)	35.1 (12.2–68.7)	125.2 (56.9–211.7) *
Free PCS (μg/dL)	217.6 (82.7–355.5) *	39.3 (8.7–98.5)	56.2 (11.4–122.6)	65.4 (12.5–149.7)	214.1 (52.2–464.3) *
Free PhS (μg/dL)	56.8 (31.6–127.2) *	18.6 (9.7–57.0)	22.8 (12.4–74.1)	32.1 (15.7–81.7)	84.4 (62.5–221.8) *
Free IAA (μg/dL)	17.6 (12.8–26.2) *	3.8 (1.8–6.5)	5.3 (2.3–9.1)	7.3 (3.2–11.1)	22.0 (12.0–33.3) *

IAA, indole acetic acid; IS, indoxyl sulfate; PCS, p-cresyl sulfate; PhS, phenyl sulfate. * $p < 0.01$ vs pH 8.4.

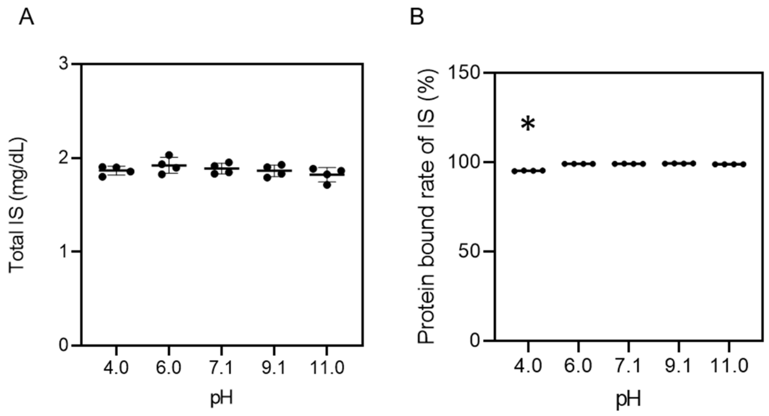

Figure S1. Protein-unbound and -bound indoxyl sulfate (IS) at pH 4–11 in the presence of albumin. pH of albumin solutions (3 g/dL) were adjusted to 4.0, 6.0, 7.1, 9.1, and 11.0, and IS (2 mg/dL) was added to each albumin solution. Concentrations of total form IS (**A**) and protein bound rate of IS (**B**) were measured. Data are means ± standard deviation of four independent experiments. * $p < 0.01$

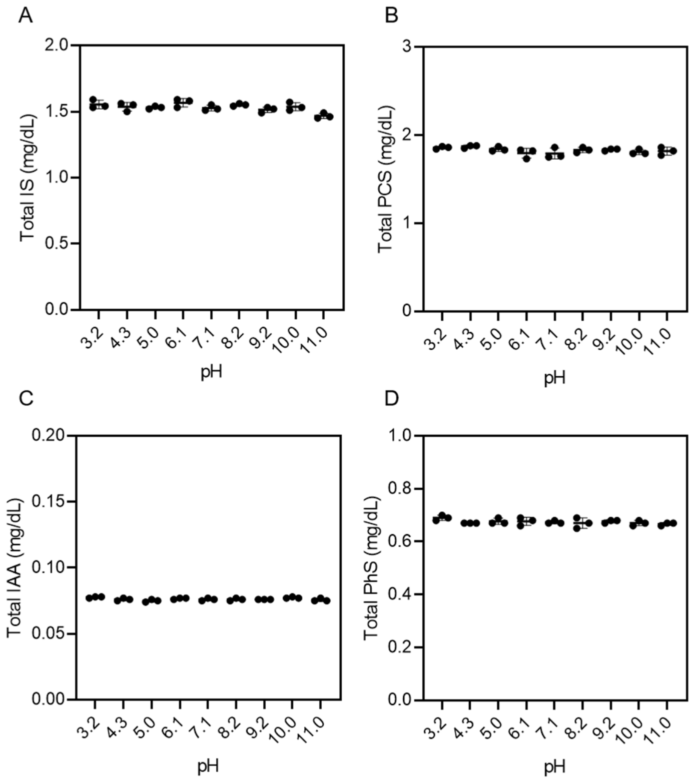

Figure S2. Protein-unbound and -bound uremic toxins at pH 3.2–11.0 in human serum. Human serum samples from non-CKD subjects containing : (**A**) indoxyl sulfate (IS), (**B**) p-cresyl sulfate (PCS), (**C**) indole acetic acid (IAA), or (**D**) phenyl sulfate (PhS) at the concentrations of typical patients undergoing dialysis were adjusted pH at 3.2 to 11.0. Concentrations of total form IS were measured. Data are means ± standard deviation of three independent experiments. CKD, chronic kidney disease; IS, indoxyl sulfate; PCS, p-cresyl sulfate; IAA, indole acetic acid.

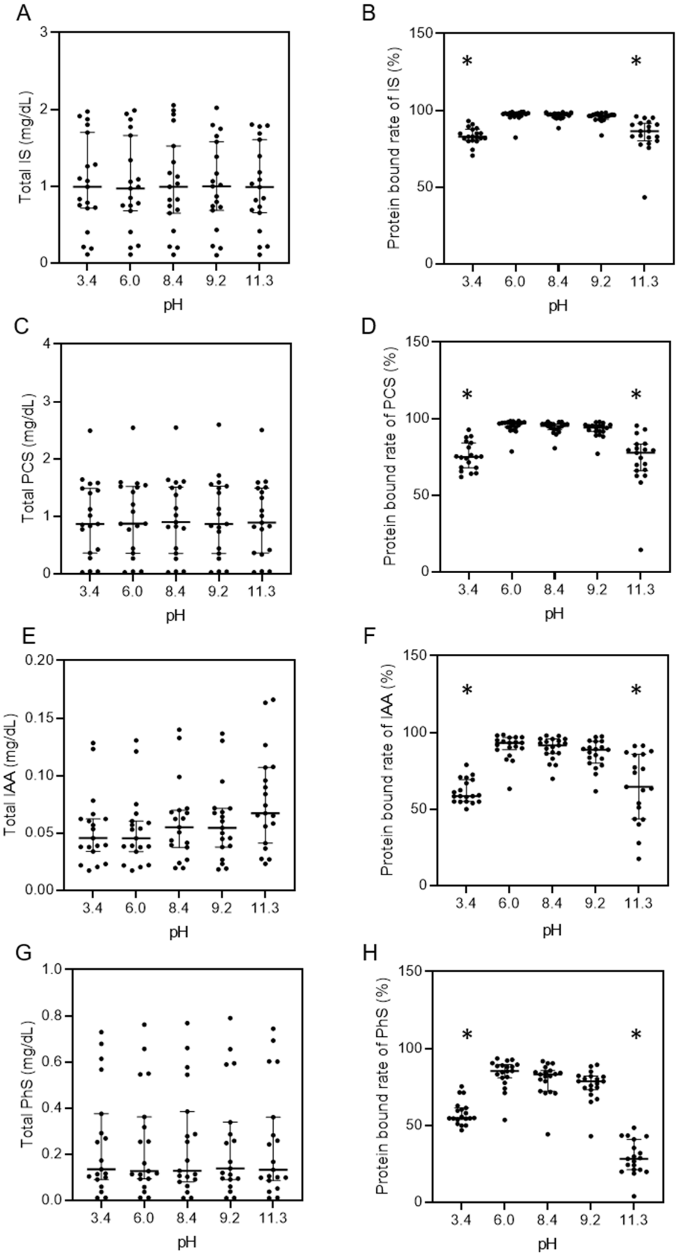

Figure S3. Protein-unbound and -bound uremic toxins and its protein bound rate at pH 3.4–11.3 in serum from patients undergoing hemodialysis. Serum levels of total form and protein bound rate of IS (**A** and **B**), PCS (**C** and **D**), IAA (**E** and **F**) and PhS (**G** and **H**) at each pH were measured. Data were expressed as medians (interquartile range) ($n = 19$). IS, indoxyl sulfate; PCS, p-cresyl sulfate; IAA, indole acetic acid; PhS, phenyl sulfate. * $p < 0.01$ vs. pH 8.4.

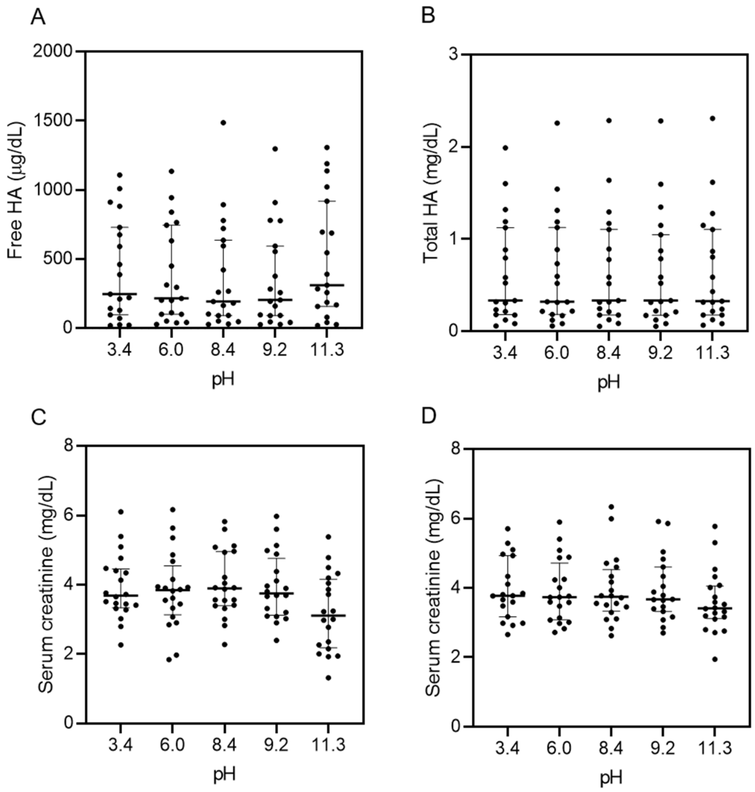

Figure S4. Hippuric acid at pH 3.4–11.3 in serum from patients undergoing hemodialysis. Serum levels of free (**A**) and total (**B**) fraction of hippuric acid (HA) and creatinine before (**C**) and after (**D**) centrifugation at each pH were measured. Data were expressed as medians (interquartile range) (*n* = 19).
